# Music Therapy as Treatment of Negative Symptoms for Adult Patients Diagnosed with Schizophrenia—Study Protocol for a Randomized, Controlled and Blinded Study

**DOI:** 10.3390/medicines6020046

**Published:** 2019-04-01

**Authors:** Inge Nygaard Pedersen, Lars Ole Bonde, Niels Jørgensen Hannibal, Jimmy Nielsen, Jørgen Aagaard, Lars Rye Bertelsen, Silvia Beatriz Jensen, René Ernst Nielsen

**Affiliations:** 1 Department of Communication and Psychology, Aalborg University, 9000 Aalborg, Denmark; lobo@hum.aau.dk (L.O.B.); hannibal@hum.aau.dk (N.J.H.); 2 Department of Psychiatry, Aalborg University Hospital, 9000 Aalborg, Denmark; Jimmy.nielsen@regionh.dk (J.N.); jaagaard@privat.dk (J.A.); larb@rn.dk (L.R.B.); silvia@musikterapeuterne.dk (S.B.J.); ren@rn.dk (R.E.N.); 3 Department of Clinical Medicine, Aalborg University, 9000 Aalborg, Denmark

**Keywords:** schizophrenia, negative symptoms, isolation, music therapy treatment

## Abstract

**Background:** Three Cochrane reviews show that music therapy has a positive effect on schizophrenia concerning general functioning and positive/negative symptoms. This study aims to replicate these results in the Danish health system, a requirement for recommendation in guidelines from the Danish National Board of Health. **Methods:** The study is a randomized, controlled multi-site study, with a blinded design, aiming to include 90 participants who are 18–65 years in age, diagnosed according to ICD-10 with a schizophrenia diagnosis. The participants are randomized to one of two different music therapy activities for 25 weekly sessions. The study interventions are added to standard care. Outcome measures are rated at baseline, after 15 sessions and post therapy. A qualitative interview is performed as a one month follow up at the end of study. The primary intended outcome is a reduction in negative symptoms. The secondary intended outcome is progression in quality of life, alliance and psychosocial functioning. **Results:** As this study is still running, the results are not yet available. **Conclusion:** The study will investigate the direct effects of music therapy on negative symptoms as part of schizophrenia in a blinded, randomized trial. If proven effective, music therapy can be added to the small treatment armamentarium of effective therapies for negative symptoms in patients with schizophrenia.

## 1. Introduction

### 1.1. Background

The background for this study is based on three factors: (1) The treatment possibilities for the population under investigation are poor; (2) National Boards of Health in different countries recommend music therapy differently as a treatment possibility; (3) Music therapy has shown positive results in previous studies, where music therapy was added to standard care and compared to standard care alone. All three factors indicate that more evidence-based research is needed, with an active comparator as the second arm within the field.

The World Federation of Music Therapy (WFMT) produced the first common definition in 1996, and the most recent version from 2011 reads as follows: “Music therapy is the professional use of music and its elements as an intervention in medical, educational, and everyday environments with individuals, groups, families or communities who seek to optimize their quality of life and improve their physical, social, communicative, emotional, intellectual and spiritual health and wellbeing. Research, practice, education and clinical training in music therapy is based on professional standards according to cultural, social and political contexts” [1].

#### 1.1.1. Treatment Possibilities

Negative symptoms of schizophrenia have gone from being described as the primary feature of schizophrenia to being a secondary phenomenon [2]. Negative symptoms have been correlated with a lower quality of life, lower overall social and global functioning, as well as being associated with a decline in cognitive function [2]. Despite the correlation between negative symptoms and key areas of living, few studies have investigated possible treatments for the symptom cluster, and even fewer have showed an effect in the treatment thereof [3].

#### 1.1.2. Recommendation of Music Therapy for the Population

Previous studies have shown that music therapy can be an effective psychosocial treatment approach [4,5,6,7]. Based on these studies, art therapy, including music therapy, has since 2014 been recommended as a treatment method for patients diagnosed with schizophrenia in the National Institute for Health and Care Excellence (NICE) guidelines (UK) to promote recovery. It is stated that “...art therapies should be recommended to all people with psychosis or schizophrenia, particularly for the alleviation of negative symptoms.” [8].

The NICE guidelines justify their recommendation by stating that people with schizophrenia should be enabled to experience themselves differently, to be offered new ways of relating and expressing themselves and organizing their experiences into a satisfying aesthetic form ([8] p. 593).

The Royal Australian and New Zealand College of Psychiatry claimed in 2016 that the evidence from randomized, controlled trials in music therapy is inconclusive. Therefore, more studies investigating the effects of music therapy in this patient group are warranted before music therapy can be recommended as a standard treatment [9].

In Norway, the national treatment guidelines from (Helsedirektoratet) state that music therapy promotes wellbeing and that the treatment should start as early as possible in order to reduce negative symptoms (our translation) [10].

In Denmark, music therapy is not recommended in the treatment guidelines for schizophrenia in The Danish National Board of Health. It is recommended to “...develop and prove more effective psychosocial evidence-based treatment methods for this population” (our translation) ([11], p. 51).

This study is an attempt to contribute to the development of evidence-based treatment methods.

#### 1.1.3. Previous Positive Results

Three Cochrane reviews [4,6,7] have shown the positive effect of music therapy in patients diagnosed with schizophrenia, with an improvement of several general symptoms (among others, attention and motivation) and a reduction of negative symptoms. The main intervention methods of the studies have been (1) musical improvisation (where the music therapist plays a frame around the expression of the client and thus helps provide a musical structure where the client can feel like being a real part of interplay) and (2) song-writing (where the music therapist helps the client to structure texts made by the client and create a melody and musical accompaniment so they can sing the song, based on the text by the client, together. The studies were based on humanistic, resource-oriented and psychodynamic approaches. In all studies included, music therapy was added to standard care and compared to standard care alone. These reviews do not examine which elements in music therapy contribute to the overall effects observed. Qualitative research in the form of case studies has elucidated which elements in music therapy are assumed to be effective in the treatment. Musical elements, as well as elements in the therapeutic relationship seem to be crucial. Basic communication elements such as turn-taking in giving and taking a tone, a rhythm or a small melody are assumed to be effective in musical communication [12,13,14]. Additionally, joint musical expression at a very slow tempo, with as many repetitions as needed, are assumed to be effective in musical interplay [15,16]. These communication forms would not be as effective or meaningful in verbal communication. Furthermore, patients report it is meaningful to play or vocalize simultaneously with the therapist when the therapist follows the expression of the patient either in the form of imitation, matching or affective attunement [17]. These mechanisms are not so accessible in verbally mediated treatment.

Research on music and the brain has shown that the perception of music indicates that music and specifically music listening can positively affect brain chemistry. Among other effects, music listening can strongly affect and change activities in brain areas connected to emotional regulation and social response, such as the limbic and paralimbic structures [18]. This is in line with research showing that listening to preferred or selected music in the form of playlists can regulate emotions and moods in different ways [19,20]. Finally, qualitative research based on the analysis method of grounded theory in music therapy in patients diagnosed with schizophrenia, or those who present schizotypical disorders, has shown improvements in music listening, where the focus of the individual evokes inner imagery which can be shared verbally with a group of clients and the therapist [21]. Smaller studies, where mutual music improvisation among the therapist and client in a 1:1 setting was the applied intervention, also point at the importance of how the music therapist is consciously present in the therapeutic relationship when working with this population [22,23,24,25]. A general focus in these studies is on the therapist’s listening attitude and the therapist’s consciousness of being present in a disciplined state of being. Here, the therapist identifies with the patient’s state of being by consciously imitating and following the state of being of the patient, as expressed through musical interplay in the beginning. Gradually, with the right timing, the therapist can start to differ in musical expressions through small variations in musical expressions, gradually moving to a more inter-subjective state of being together, where both are engaged in the interplay as two subjects playing together [15,24,26,27].

Based on these factors, the aim of this study is to investigate whether music therapy can reduce negative symptoms in patients diagnosed with schizophrenia by using a randomized, controlled and blinded study design with an active comparator for the interventions. Furthermore, the study aims to examine if individual music therapy can increase the quality of life and establish progression in the alliance in the therapeutic relationship. Lastly, the study aims to investigate if the intervention can support and improve psychosocial functioning.

### 1.2. Hypothesis

We hypothesize that participants who are randomized to group I (the experimental group), when performed by educated and experienced music therapists offering multiple music therapy techniques adapted to the needs of the participant, will have a larger reduction in negative symptoms as measured by the clinical rating scale than participants who attend group II (the control group), where music is performed by non-music therapists, offering solely music listening. Furthermore, we hypothesize that participants randomized to group I will experience a larger increase in quality of life and in the progress of alliance building and psychosocial functioning as compared to group II.

## 2. Materials and Methods

### 2.1. A Randomized, Controlled and Blinded Study

The examination is a randomized, controlled, multi-site study, applying a blinded design including 25 sessions of manualized music therapy for the intervention group (group I) and 25 sessions of manualized music listening to selected playlists for the second arm with an active comparator (group II). The number of sessions is based on research concerning the dose response effect for music therapy for people with serious mental disturbances [28]. The arms of interventions are called intervention I and intervention II in the present study. Participants are rated at baseline, after 15 sessions and after 25 sessions post-therapy. At follow up (four weeks after post therapy at the end of study), a short, semi-structured interview will be performed with all participants. (See Table 1). Before baseline, all participants are informed (1) that they will be offered 25 sessions of music therapy, (2) that two different activities will be tested and compared (3) and that those performing the sessions are all referred to as therapists and are familiar with being together with people diagnosed with schizophrenia. (4) The participants will also be informed that recording of the musical activities might occur. All sessions will take place in the same equipped music therapy rooms. Intervention I is carried out by trained and experienced music therapists who are familiar with the population under examination, being carefully trained in and following a manual developed for this intervention in the study. Professional music therapists in Denmark all have a five-year full-time university BA/MA training, including several internship periods [29]. This training is based on a psychodynamic, humanistic and neuropsychological theoretical platform and is structured in three parallel running study tracks with equal weight through all five years. These tracks include an academic theoretical study track, a music-based improvisational methodological study track and a psychotherapeutic, self-experiential and clinical study track [29]. Due to the manual for Group I several music therapy methods and techniques can be applied including active methods (where the therapist and participant play or sing together, improvise, write songs or move to music), as well as receptive methods (where the therapist and participant listen to music together, either in the form of playlists selected by the music therapist, or in the form of participant-chosen or therapist-chosen music). The focus of the manual is about perspectives on the therapist’s way of being present and positions in the therapeutic relationship.

Intervention II is carried out by non-music therapists who are familiar with the population under examination, being carefully trained in and following a manual developed for this intervention in the study. Intervention II includes solely listening to specific playlists developed by music therapists. (This intervention is similar to one of the possible intervention methods in intervention I and thus both intervention forms can be called music therapy activities). All therapists performing intervention II are trained how to apply the playlists by a music therapist. Therapists performing intervention I have, as a premise, the possibility of applying multiple music therapy techniques, including the selected playlists. Their choice of techniques is currently adapted to the needs of the participants. Both manuals are developed according to unique, essential, possible and not-possible/proscribed principles for interventions and for the attitudes of the therapist in work with this population [30,31].

All participants included in the study and clinical personal accessing the participants will be blinded. Therapists, investigators and other researchers in the research steering group, including those who make the qualitative analysis as part of the data collection, will not be blinded. The participants will be rated with the scales and measures listed below in Section 2.4.

For analyzing musical communicative capacities, only those attending intervention I will additionally be subject to audio recordings of musical improvisations in sessions 1, 15 and 25 (if improvisation is performed in those sessions), as musical improvisation is not a part of intervention II. Finally, the therapy session reports about the participants’ experiences of music listening, experiences of music performance and level of initiative in verbalization will support the results as a whole.

The study involves two regions in Denmark: The North Jutland Region and the Capital Region. Within these regions, nine municipalities are involved. A cooperation agreement is signed for each region and for each of the municipalities.

### 2.2. Recruitment Processes

In the recruitment process, administrative and/or research heads of psychiatric centers will be contacted, and information meetings for daily contact persons for potential participants will be carried out at all centers that show interest in cooperation. Posters and leaflets will be spread throughout the centers and further information meetings will be offered if needed. These meetings often take place in two steps. The first step includes meeting with the contact person(s) alone and the second step is meeting with the contact person(s) and those diagnosed with schizophrenia, who have shown interest and who are supposed to be potential participants. After a trial period of five months, we learned that in each center, a contact person should be appointed to remind staff members to refer and to collect referrals and forward these with the needed information to the research coordinator. The coordinator receives the name, telephone number and e-mail address of the participant through secure mail. Both the potential participant and the contact person are permanently in contact with the coordinator (the same person for all participants in respectively the Region of North Jutland and the Region of the Capital) about potential questions around participation. The coordinator arranges for the inclusion screening procedure. At this first screening procedure, the project nurse asks the participant to sign an informed consent form in order to provide allowance to receive information of diagnosis, medical information and the hospitalization history of the single participant. If this is not possible, the client cannot be included in the study. The informed consent for taking part in the project as a whole is also signed at the first screening procedure after further information of the conditions involved in the study. After randomization, the coordinator will connect the participant and a relevant therapist, scheduling sessions at the equipped music therapy clinic room, located as close as possible to where the participant is living. If the participant is unable to take public transport, taxi tickets can be offered. The coordinator takes care that a time schedule for 25 sessions is made by the therapist and accepted by the participant. The therapist receives information of the name, telephone number and email address for both the participant and the contact person. The coordinator registers these sessions using Google Calendar. He is the person who will coordinate all screening procedures and interviews throughout the participation period of the single participant, due to the rules of the research design. All information about the single participant will be saved on a secure electronic drive.

The coordinator ensures that all relevant information is available and coordinates the inclusion screening procedure between the participants and the project nurse (screener). If the screening procedure results in inclusion, the participant is randomized by the Hospital Pharmacy, Region of Northern Jutland, which is not otherwise involved in the present study. A random variable block size randomization will be utilized in the study. The first session will take place no later than 14 days after the screening procedure to ensure the validity of the positive and negative syndrome scale (PANSS) evaluation conducted at the first screening. The study coordinator is responsible for assigning a relevant therapist, scheduling sessions and booking session rooms.

### 2.3. Inclusion and Exclusion Criteria

#### 2.3.1. Inclusion Criteria

The inclusion criteria are as follows:

(1) Age 18–65 years;
(2) Diagnosis of schizophrenia by ICD-10 (F20);
(3) PANSS negative subscale, minimum ≥4 on two of the following items, blurred affect (N1), emotional withdrawal (N2), poor contact (N3), passive apathetic social withdrawal (N4) and lack of spontaneity and flow in dialogues (N6).

#### 2.3.2. Exclusion Criteria

The exclusion criteria are as follows:

(1) Diagnosis of schizophrenia less than two years ago;
(2) Patients hospitalized less than three months ago due to their psychiatric illness;
(3) PANSS positive subscale (P1–P7) > 28;
(4) Patients who have had changes in psychotropic treatment during the last month;
(5) Significant alcohol/drug abuse that may interfere with study participation, as judged by the investigator;
(6) Presence of secondary negative symptoms, defined as:
  (a) Calgary Depression Scale for Schizophrenia (CDSS) score >7 or;
  (b) Neurological side-effects UKU score >1 in the following items: Dystonia (2.2), rigidity (2.3), hypokinesia/akinesia (2.5), tremor (2.6) and motor akathisia or;
  (c) Sedative side-effect UKU item (1.3) or a sleepiness score >1;
  (d) Patients who have attended individual music therapy within the last 2 months.

#### 2.3.3. Treatment Schedule and Withdrawal Criteria

The treatment schedule is constructed to offer weekly sessions of 50 min of music therapy activities each 7 day +/− 1 day, resulting in a minimum of five days and a maximum of nine days between sessions. A session plan of 25 sessions is scheduled before the start of intervention and is accepted by the participant. If the participant fails to meet up to any of the scheduled sessions, with or without notice, the session will not be replaced. If the therapist cancels any of the scheduled sessions, replacements will be offered. The 25 scheduled sessions exclude holiday weeks. Participants will be withdrawn from the study as a whole if more than 30 days pass between two sessions or if the participant fails to turn up for more than five sessions. The participant will be reminded and warned by telephone if they fail to appear at three and four of the scheduled sessions, with a reminder of exclusion from further participation if a total of more than five lacking appearances occur. They will also be reminded if more than three weeks between scheduled sessions pass, often as part of a holiday, resulting in it being mandatory to attend the week after the holiday. Each therapist in the study is not allowed to plan for more than three combined weeks of holiday during each treatment schedule. Each time the participant fails to turn up without notice, the therapist will contact the participant to ensure the participant is safe and has planned not to attend this session. The participant will also be withdrawn from the study if they withdraw their informed consent. If hospitalization occurs during the study, the participant can continue in the study as long as the duration is short term (<5 days) and no change in medication has occurred.

Withdrawing from the study will in no way affect the individual participants’ treatment as usual.

### 2.4. Outcomes

**Figure 1:**
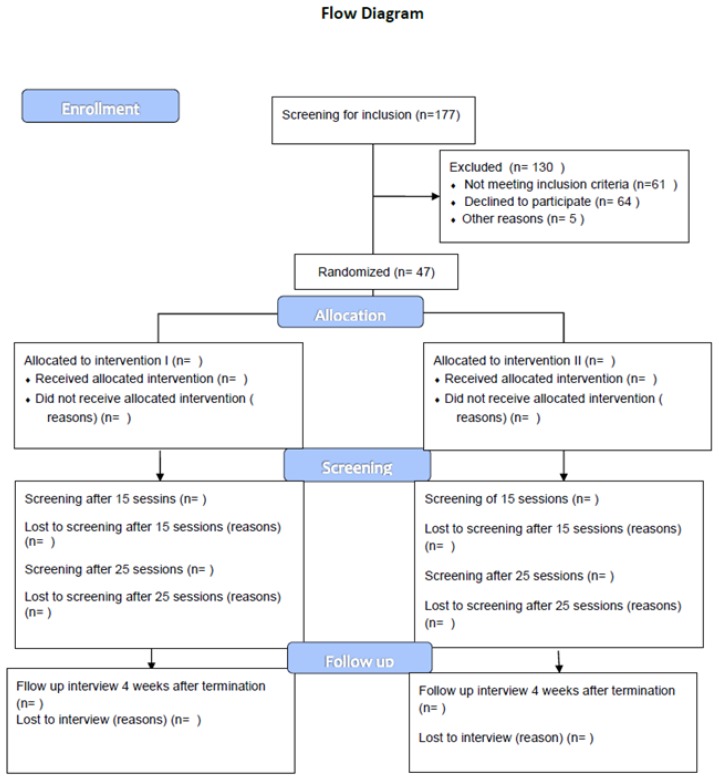
Flow diagram of the experiment design. As the recruitment process is currently running, only the inclusion data can be presented.

The outcome measures include primary and secondary outcomes. The primary outcome measure is a change in the PANSS negative subscale from baseline to the end of the study. The secondary outcomes are a change in Brief Negative Symptom Scale (BNSS), measurement of quality of life, the capacity of building an alliance, global functioning and for the experimental group, musical communication ability from baseline to the end of the study (see Figure 1).

#### 2.4.1. Negative Symptom Measurements

As we are investigating the effect of music therapy on negative symptoms in patients with schizophrenia, we wished to evaluate this symptom domain as carefully as possible. When measuring negative symptoms, symptoms can be divided into primary and secondary negative symptoms. The primary symptoms are the core negative symptoms which are not a consequence of, for example, the side-effects of drugs, depression or a lack of social activity. As a result, we wished to have a measure of negative symptoms (PANSS and BNSS), as well as measure symptom domains which can present themselves as negative symptoms. As a result, we have chosen to measure depressive symptoms (CDSS) and medication side-effects (UKU) in addition to the scales that measure negative symptoms specifically.

The Positive and Negative Syndrome Scale (PANSS) consists of seven items for positive symptoms, seven items for negative symptoms and 16 items for the evaluation of general psychopathology. The PANSS is validated and verified and is standard in effect studies of antipsychotics [32,33]. The Structured Clinical Interview-PANSS (SCI-PANSS) is a structured interview designed to help the interviewer in getting the information needed to perform the rating. The SCI-PANSS will be used in the study [33,34,35]. Interrater reliability has been shown to be good on the full PANSS scale score, with intraclass coefficients of 0.98–0.99, with an acceptable interclass coefficient of 0.83–0.90 when using the SCI-PANSS interview [36]. Part of the PANSS rating is video recorded and kept in the data collection, as to furthermore enable analyses of interrater reliability.

The Brief Negative Symptom Scale (BNSS) consists of 6 subscales and 13 items on anhedonia, distress, asociality, avolution, blunted affect and alogia. The advantage of the scale is a separation of appetitive and consummatory anhedonia, asociality and internal experience. It has a threefold aim of examination (knowledge of behavior, of the social context and of the report of the experience of the participant concerning everyday life) [37].

#### 2.4.2. Quality of Life and Capability of Attachment Measurements

WHOQOL-Bref (Quality of Life) is a self-reported questionnaire containing 26 items. Each item is rated on a Likert-type scale ranging from zero to five, where zero is very bad and five is very good [38].

The Helping Alliance Questionnaire (Patient Version) (Haq-II) is a self-reported questionnaire containing 19 items rated on a Likert-type scale with a score from zero to six, spanning from do not agree at all to total agreement [39]. The measurement tool is important to investigate if a potential reduction of negative symptoms may increase the ability of building alliances. A former research project showed positive results in the increase of alliance building in music therapy treatment in a study population including patients diagnosed with schizophrenia [40].

#### 2.4.3. Measuring Secondary Negative Symptoms

The Calgary Depression Scale for Schizophrenia (CDSS) is used as a scale of depression for schizophrenia. Depression symptoms can be difficult to differentiate from the negative symptoms seen in schizophrenia. The CDSS consists of nine items. The CDSS is reliable and valid for the evaluation of depression in schizophrenia, and in differentiating depression from negative symptoms when combined with PANSS [41]. Each item can be given a score between 0 and 3, which gives a total score between 0 and 27. Light depression is defined as a total score ≥3 but <7, moderate depression is defined as a total score ≥7 but <11 and severe depression is defined as a total score ≥11.

UKU is an acronym for the Danish name “Udvalg for Kliniske Undersøgelser” (Task Force for Clinical Investigations). The UKU side-effects scale is used for a general assessment of side-effects induced by treatment with antipsychotics. The UKU scale is divided into four main areas: Mental, neurological, autonomic and other side-effects, containing a total of 48 items rated by the clinician [42].

#### 2.4.4. Measuring State of Functioning

The Global Assessment of Functioning (GAF) scale is a clinical evaluation of the participant’s overall function levels. Generally, it includes psychological, social, interpersonal and occupational functioning in regard to mental-health illness. The scale ranges from 0–100, with a higher number indicating superior functioning [43].

#### 2.4.5. Measurement of Musical Communication Ability

The Musical Interaction Rating Scale (MIR(S)) is applied solely for participants in group I if musical improvisation takes place in sessions 1, 15 and 25. It is a non-standardized tool specifically created for music therapy to analyze and rate musical interactions (the musical contact) between the participant and the therapist. Ten items are rated, ranging from no contact to fluent contact between the therapist and participant [44,45,46].

#### 2.4.6. Other Measurements

For each session, a detailed therapy session report is filled in. Different versions of the reports are available depending on which group the participant is randomized to. The report documents attendance, choice of techniques by the therapist, choice of music by the therapist or the participant, and finally the musical and verbal initiative of the participant.

At the four weeks follow-up, a semi-structured interview will take place, performed by the same researcher for all participants. A four-week timespan was chosen as the participants most commonly are chronically ill, and cognitive deficits could result in recall bias.

The interview is structured in two parts. The first part consists of short questions about self-experienced progressions concerning the participant’s work situation, family and social relationships and activities and personal issues (self-reliance concerning hygiene and cleaning). The second part consists of open questions about the participant’s experiences of joining the music therapy activity sessions. To create a safe setting around this interview, it is important that there is sufficient time and that it is performed where the participant wants the session to take place (e.g., at the participant’s private home, at a bench close to home, in the same room as the music therapy activities have taken place or at the place of the screening procedures).

### 2.5. Data Collection and Management

#### Case Report Form and REDCap

All data from the screening procedures will be collected in a case report form for each participant, excluding the semi-structured qualitative interview data and session schedule data. All data collected will further be entered into a REDCap electronic database, utilizing double entry to minimize the risk of errors. All information from the case report forms will be stored by the Aalborg University Hospital Psychiatry Department. When screening procedures take place on sites away from the hospital, the screening data of each case will be kept in a client project report, which is held securely until the project leader transports the data files to the hospital where they are entered into REDCap.

Data from semi-structured interviews and session schedules will be stored by The Music Therapy Clinic, an integrated institution between Aalborg University and the Aalborg University Hospital Psychiatry Department. Therapy session reports are sent by each of the therapists immediately after each session by secure mail to the coordinator, who saves the files at a secure electronic drive. Qualitative interviews are kept by the interviewer in a locked closet until the study is finished and the data have to be analyzed. These data are not entered into REDCap.

### 2.6. Ethical Approval

#### Different Approvals

This study protocol is approved by The Regional Committee on Biomedical Research Ethics, Region of Northern Jutland, Denmark (ID N-20150054) and the Data Protection Agency of Denmark (2015-144). The study is performed in accordance with the Declaration of Helsinki. Written informed consent forms will be obtained before participant inclusion in the study. A detailed signed cooperation agreement between the university and the hospital is developed for this study and a signed agreement from the heads of psychiatric centers from each community will be required before any recruitment can be initiated. The study is registered in TrialRegistration.gov (ID number NCT02942459) registered retrospectively since October 2016.

### 2.7. Sample Size

#### Estimation of Sample Size

Utilizing previous data, we estimated an effect size of 0.6 (medium effect according to Cohen’s d). With a selected level of significance of 0.05 and a power of 0.8, a power calculation performed in R with a conservative approach (applying a two-sided *t*-test with two samples), resulted in each group having to count a minimum of 44.5 persons. The data utilized in the power calculation did not use an active comparator, which might result in a lower effect size in the current study. We furthermore estimate a drop-out of 10–20% of participants, resulting in an estimated required sample size of 60 persons in each group.

## 3. Results

### 3.1. Statistical Analysis

#### 3.1.1. Form of Statistical Analysis

As the study is still running, no final results are available. The results will be generated through statistical analysis where the endpoints from PANSS, BNSS and WHOQOL-BREF will be analyzed using mixed model effect analysis. Predictors for the response to the treatment will be performed by multiple logistic regression, using the severity of psychiatric disorders, timespan from diagnosis identified, psychotropic medication, PANSS score results and regional contra communal treatment as usual, as explanatory variables with a regression model being performed for each of the two randomized groups. The analysis will focus on identifying predictors of improvement of negative symptoms and change of quality of life, alliance and global functioning, as well as predictors for improvement connected to treatment (music therapy activities I or II). Both per protocol and intention-to-treat analyses will be performed. Intention to treat is defined as utilizing data from all participants, even if the single participant has not attended all 25 sessions. At the time of discontinuation from the study, a final assessment will be conducted, if possible. Participants will not be followed up on after this time point.

All tests will be applied double sided and the significance limit is defined as *p* < 0.05.

#### 3.1.2. Analysis of Compliance

A sensitivity analysis will compare compliance versus non-compliance. Compliance will be calculated based on the number of sessions received divided with number of sessions offered and multiplied by one hundred.

#### 3.1.3. Qualitative Analysis

During the semi-structured follow-up interviews, the interviewer will write down each word expressed by the participant. Most participants are rather short of words and need time to formulate their experiences. Each word in the written material is read out loud for the participant at the end of the interview to ensure the participant agrees to what is reported and that they accept the text. This procedure replaces member checking with this population, as audio recording mostly has a disturbing effect, and as they might have difficulties in remembering what they expressed, having a written text for several weeks later is very helpful. The texts will be analyzed through phenomenological analysis and will add to the results in the form of short theme-based narratives on developments in work situation, social and relational issues and patient reported overall experiences of participation in the study. Additionally, music improvisation analysis will add to the results concerning progression in levels of contact in the musical interplay of the therapeutic relationship.

## 4. Discussion

### 4.1. Blinded Design as an Innovation

This study is the first study in music therapy in patients diagnosed with schizophrenia using a randomized, controlled and blinded design. There are numerous studies and Cochrane reviews showing the positive results of music therapy on global functioning, including negative symptoms in patients diagnosed with schizophrenia where music therapy was added to standard care, comparing this to standard care alone [4,6,7].

This study will investigate if negative symptoms can be reduced when participants are offered music therapy performed by a professional music therapist, as compared to an active comparator where music listening to selected playlists is performed by a non-music therapist. The design adjusts for the Hawthorne effect, which may positively bias results of an intervention when only comparing this to standard care. This study will only capture the immediate effect on negative symptoms (if any) of the intervention. A four week follow-up includes solely a qualitative interview (see Section 2.4.6), where the subjective experiences of the participants can be unfolded. The timespan for this follow-up is rather short and could be claimed as a limitation of the study. As mentioned above, the four weeks timespan was chosen as the participants most commonly are chronically ill and cognitive deficits could result in recall bias.

#### 4.1.1. Challenging in Blinding

We are aware that the blinding of participants, as performed in this study, cannot be compared to a placebo blinding, as the intervention is a face to face procedure, and the therapists performing the interventions are not blinded.

#### 4.1.2. Challenges of the Design

We are also aware that applying a design as close to a biomedical design as possible, in an examination of a population with a complex disease and with low motivation for participation, is ambitious. We are prepared for challenges. We plan to cooperate with relevant staff members and relatives concerning motivation, attendance and other experienced challenges by the participants.

## 5. Conclusions

### Reduction in Sample Size Due to Challenges

The trial status is currently ongoing. Recruitment started in March 2016 and will continue until the end of May 2019, with the expected last participant visit in December 2019. Due to low rates of recruitment, a total of 47 participants have been included by November 2018, from the 177 referred to the study (see Figure 1). We currently aim to include a total of 60 participants. Concerning blinding, we have currently been informed that three participants receiving intervention II have expressed that they know they are not receiving music therapy due to previous experiences with music therapy. So they are not blinded. This will be transparent in the analysis of the data. In spite of challenges concerning motivation and attendance, many participants have so far been able to attend all sessions offered. The results of the current trial will be widely disseminated at national and international scientific conferences and in further scientific articles.

## Figures and Tables

**Table 1:** A SPIRIT diagram on screening procedures.

	Screening	Baseline		After Fifth before Sixth Offered Session	After Fifteenth before Sixteenth Offered Session			Less Than Eight Days after the Last Session Termination ^1^			Follow up 4–5 Weeks after Termination
No of visit	1	2		3	4			5			6
Performed by ^2^			MT/T	RP	PN	MT	RP	PN	MT	RP	RP
Information and consent	x										
Demographical data ^3^	x										x
Status of medicine	x	x ^4^			x			x			
History of abuse	x	x ^4^			x			x			
History of music therapy ^5^	x	x ^4^									
Inclusion and Randomization		X									
PANSS	x	x ^6^			x			x			
Brief Negative Symptom Scale (BNSS)	x	x ^4^			x			x			
WHOQOL	x	x ^4^			x			x			
Calgary Depression Scale for Schizophrenia (CDSS)	x	x ^4^			x			x			
Udvalg for Kliniske Undersøgelser (UKU), otherwise Task Force for Clinical Investigations	x	x ^4^			x			x			
Global Assessment of Functioning (GAF)	x	x ^4^									
Helping Alliance Questionnaire (HAQ-II)				x			x			x	
MIRS Only experimental group I			x			x			x		
Start of weekly interventions, groups I and II			x								
Start McGlashan rating after each intervention for experimental group I			x								
Termination of weekly interventions for groups I and II									x		
Interview											x

^1^ Is not performed if screening procedure after fifteenth session but before sixteenth offered session is performed ≤14 days earlier. ^2^ PN = rroject nurses; MT = music therapists group I, therapist group II; RP = research participant. ^3^ Sex, age, ethnicity, level of education, social and work situation. ^4^ Is not performed if performed by screening ≤28 days earlier. ^5^ Music therapy and other formalized music related offers other than trial treatment are registered. ^6^ Is not performed if positive and negative syndrome scale (PANSS) in screening is performed ≤14 days earlier.
